# Supplementary figures and images for: Deletion of endothelial KLF4 as a model for preeclampsia
Source: bioRxiv. 2026 Mar 31:2026.03.30.715448. Preprint. [Version 1] doi: 10.64898/2026.03.30.715448 (PMC13060099; doi:10.64898/2026.03.30.715448)

Supplemental Figure 1

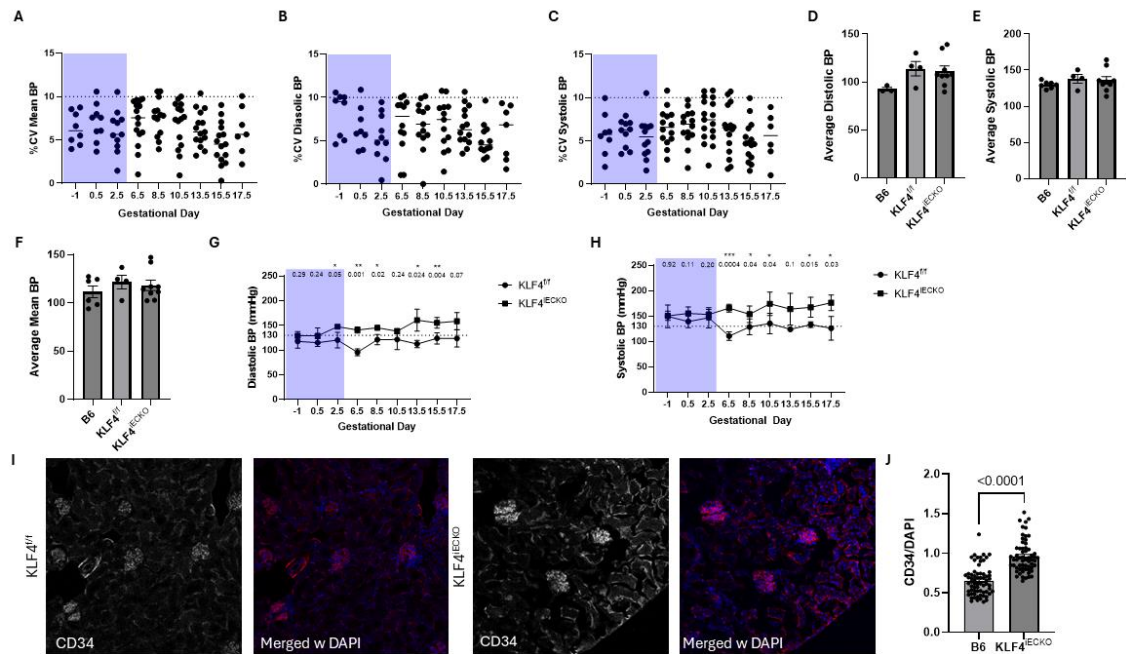

Supplemental Figure 2

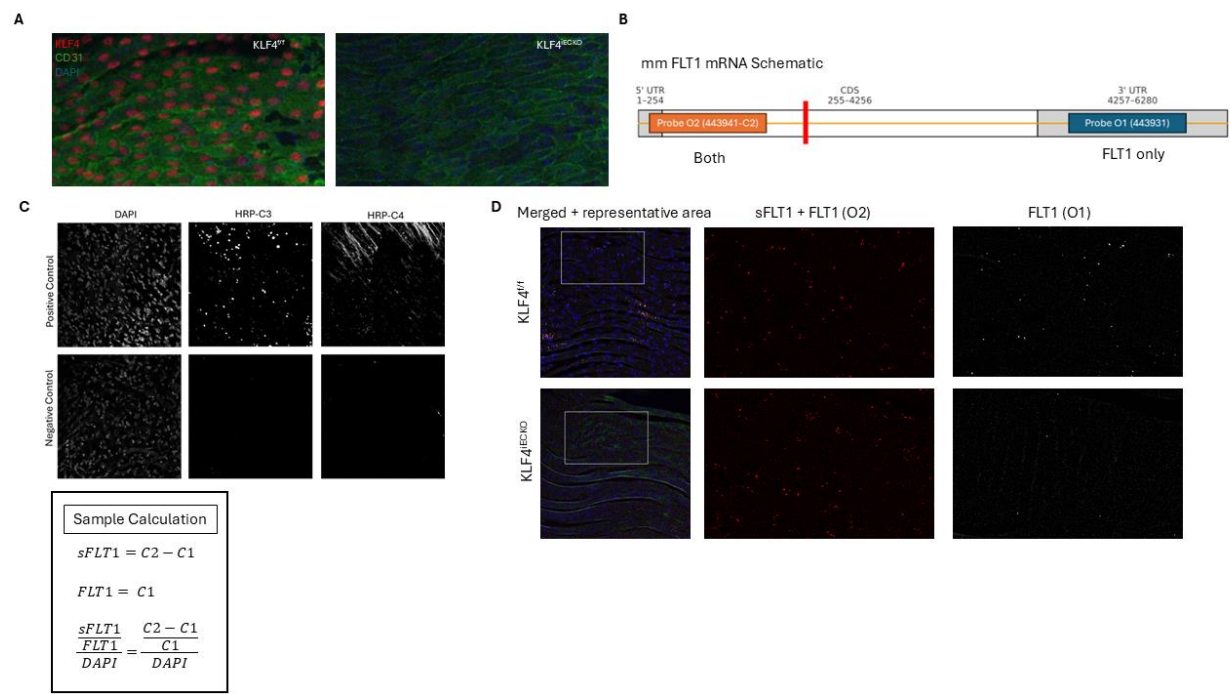

Supplemental Figure 3

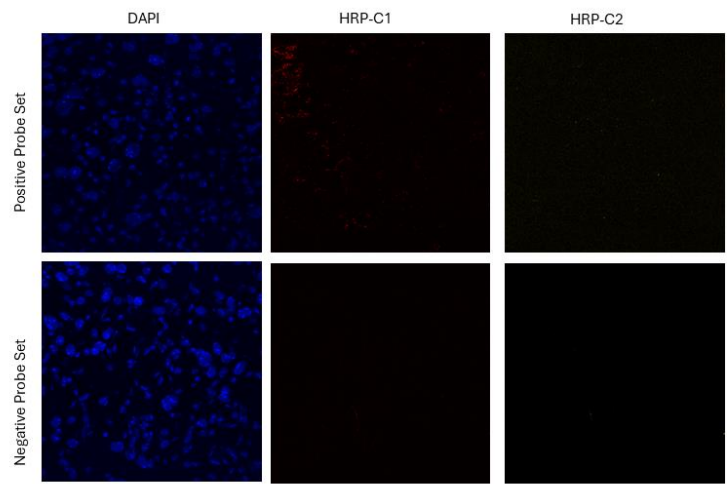

Supplement: Supplement 1 — Supplemental Figure 1. Additional assays on hypertension and kidney damage. A-C percent coefficient of variation (%CV) calculated per session per individual to ensure robust measurements. For each animal and session, BP was calculated as the mean of ≥3 accepted tail-cuff cycles. Session stability was quantified using the coefficient of variation (CV = SD/mean × 100). Cycle-level outliers exceeding ±2 SD were excluded to remove technical artifacts. Sessions demonstrating CV values within the expected physiological range (<10%) were considered stable. Days covered by purple are training days. D-F average diastolic (D) and systolic (E) and mean (F) blood pressure comparison between nonpregant B6, KLF4f/f and iECKO dams showing no difference prior to pregnancy. G-H Time course of diastolic (G) and systolic (H) blood pressure changes during pregnancy in f/f or iECKO KLF4 animals. Days covered in purple are training days. I-J Representative CD34 staining in KLF4iECKO and f/f controls with quantification (I). Supplemental Figure 2. RNAscope Schematic and Analysis. A confirmation of KLF4iECKO following tamoxifen injection. Aortas were stained en face for KLF4, CD31 and DAPI to confirm iECKO. B Mouse FLT1 mRNA schematic is shown with RNAscope probe locations and the sFLT1 i13 splice site (red line). C RNAscope probe control staining in en face aorta pieces. Laser power and exposure for each repeat experiment was determined by control probe fluorescence before imaging experimental samples. D Split channel representative RNAscope en face aortas with FN protein staining and sample calculation. Supplement Figure 3. Placenta RNAscope validation using mouse positive and negative control probes. Placenta sections from 2-3 individual litters were used to stain for positive and negative control probes. Imaging parameters (laser power, exposure) for experimental samples were determined based on detection of signal only in the positive control set, but not the negative control set, as [file NIHPP2026.03.30.715448v1-supplement-1.pdf]
